# Spatial variation of evoked potentials in porcine retinas characterized by multi electrode array upon stimulation via 3D pyrolytic carbon electrodes

**DOI:** 10.3389/fnins.2026.1808212

**Published:** 2026-05-26

**Authors:** Jesper Guldsmed Madsen, Pratik Kusumanchi, Toke Bek, Stephan Sylvest Keller, Rasmus Schmidt Davidsen

**Affiliations:** 1 Department of Ophthalmology, Aarhus University Hospital, Aarhus, Denmark; 2 National Centre for Nano Fabrication and Characterization (DTU Nanolab), Technical University of Denmark, Kongens Lyngby, Denmark; 3 Department of Electrical and Computer Engineering, Aarhus University, Aarhus, Denmark

**Keywords:** pyrolytic carbon, restoration, retinal 3D chip, retinal ganglia cells (RGC), vision

## Abstract

**Introduction:**

Visual restoration using photovoltaic retinal implants to alleviate vision loss has recently seen substantial progress. Most devices being developed are reliant upon rare earth metals, such as iridium, but their use in connection with highly immunoactive retinal tissue may pose an issue. Typical electrodes are fabricated as thin films and as such they are two-dimensional. Ideally, stimulating electrodes should be patterned into 3D topologies to optimize the electrode-tissue interface. It is currently not possible to achieve complex 3D electrode geometries with metal-based materials, which could potentially improve stimulation efficiency and resolution.

**Methods:**

This study utilizes multi electrode array analysis of the spatial variation of evoked potentials in porcine retinal explants stimulated electrically via newly developed 3D carbon electrodes.

**Results:**

Nine out of 10 explants showed significantly higher tissue activity during stimulation using the electrode, supplied with a direct current pulse with a voltage of +0.5 V typical for single-junction silicon photovoltaics.

**Discussion:**

We report no spatial biases or patterns in tissue activation. The electrode significantly activated tissue above spontaneous activity levels and did not produce spatial patterns or biases, confirming the electrode as an alternative for metallic electrodes and for further development and *in vivo* testing.

## Introduction

Artificial chip-based vision has been proposed as a possible solution to the loss of vision caused by diseases such as retinitis pigmentosa, age-related macular degeneration, and diabetic retinopathy (Palanker and Goetz, 2018; Farvardin et al., 2018; Ayton et al., 2020; Stingl et al., 2015; Holz et al., 2025; Vagni et al., 2022). As the prevalence of these diseases account for a substantial part of reduced vision globally (Flaxman et al., 2017; Wong et al., 2014), artificial restoration of vision using stimulating implants has received some attention, and some degree of success, with devices in clinical trials.

Common for much of the development of such devices is that they rely on stimulating electrodes comprised of metallic elements such as gold, platinum, titanium, and especially iridium in the composition of sputtered iridium oxide (Onnela et al., 2011; Haas et al., 2020; Barriga-Rivera et al., 2017). While noble metals due to their biocompatibility have a long history of use as material for implantation there are notable potential shortcomings for their use in retinal stimulating electrodes. First, precise dimensional control allowing for 3D structuring of electrodes is not easily possible with metallic compounds is not currently possible with metallic compounds. Secondly, noble metals are increasingly scarce. Finally, the biocompatibility of these metals, while sufficient for use in many implantation applications, may be limited when in inherent contact with neuronal tissue (Göbbels et al., 2010; Chan et al., 2021). This may especially be the case in the very immunoactive tissue of the retina. To overcome this challenge, a non-metallic 3D electrode has been developed from non-immunoreactive pyrolytic carbon and shown to be capable of evoking retinal activity (spiking) in porcine retinal tissue *ex vivo* (Kusumanchi et al., 2025) within voltage ranges possible to be delivered by a photovoltaic implant. UV photolithography and subsequent pyrolysis of SU-8 photoresist have previously been used to produce various 3D electrode geometries (Mantis et al., 2021; Amato et al., 2015; Teixidor et al., 2008; Wang and Madou, 2005) and the resulting pyrolytic carbon is promoting cell adhesion and interfacing with neurons (Amato et al., 2015; Peltola et al., 2017; Vomero et al., 2017; Asif et al., 2020).

Further testing of the concept of a non-metallic 3D carbon electrode for retinal implantation is necessary. Among others it is important to demonstrate that the electrode in the current configuration does not display any spatial biases or patterns in the evoked activity. In this study we attempt to show that when presented with a uniform and controlled direct current pulse input, the resulting stimulation of tissue across the electrode can result in spatially unbiased evoked activity. As mentioned, the future goal of the electrode is to pair it with a photovoltaic power source, which, when in use, would likely not deliver as uniform an input as a direct current pulse. As such, it is important to test for spatial biases or uneven stimulation of tissue before this pairing is done. Unbiased spatial evocation of activity is critical, as uneven stimulation can result in either blind spots (under stimulation) or white spots (over stimulation) in the visual information being passed to the visual cortex in a future implant utilizing the electrode. These considerations become even more important when the size of the electrode is decreased to form electrically isolated pixels with a pixel density sufficient to produce visual resolutions above the legal blindness limit for future retinal stimulation using photocurrent. Having shown the carbon electrodes capable of producing biological responses (Kusumanchi et al., 2025), this study thus attempts to further determine the spatial variation of the evoked activity.

When attempting to determine the spatial variation of the activity evoked by the electrode, several aspects have to be considered. The porcine tissue samples being stimulated in *ex vivo* studies will have some degree of variation in responsiveness, based on the state of each individual neuroretina sample and the eye from which it was collected. This arises from factors such as variation in time from dissection to use, dissection skill and state of the animal before slaughter. This may be termed the inter-eye-variance. Likewise, each individual retinal tissue sample may have variation in responsiveness across the sample, due to local variations of the density of responsive cells. This may be termed the intra-eye-variance. Finally, variation in the measured responsiveness of a given sample might be due to methodological factors regarding the setup used, especially factors such as variation in contact with both stimulating and recording electrodes. Taking these sources of response variation into account, it is unlikely that all stimulations of all samples will produce spatially evenly distributed and identical responses.

In this study we present an analysis of the spatial variation displayed by porcine retinal tissue stimulated by a 3D carbon electrode to determine whether the electrode could produce unbiased stimulation above spontaneous activity level. We report that the electrode was able to achieve above spontaneous tissue activity levels in eight of 10 tissue samples tested, all from individual eyes, and that no discernable spatial pattern or bias was displayed in the resulting evoked activity. The analysis utilized in this study will also be of importance in future studies where light and photovoltaics will be applied to achieve heterogenic stimulation to try and produce tissue activation in correspondence with differential input intensities, corresponding to actual visual input.

## Materials and methods

### Chemical solutions

For storage and transportation, physiological saline solution (PSS) with the following composition was used: 118 NaCl mM, 4.8 KCl mM, 1.14 MgSO_4_ mM, 25 NaHCO_3_ mM, 5 mM Hepes, 1.5 mM CaCl_2_, 5.5 mM glucose. For experiments, PSS1.6 with the following composition was used: 119 mM NaCl, 4.7 mM KCl, 1.17 mM MgSO_4_, 25 mM NaHCO_3_, 5 mM Hepes, 1.6 mM CaCl_2_, 5.5 mM glucose, 1.18 mM KH_2_PO_4_, 0.026 mM EDTA. PSS0.0 refers to PSS1.6 where CaCl_2_ has been omitted. PSS1.6 was heated to 37 °C and oxygenated by bubbling with a gas mixture of 95% atmospheric air and 5% CO_2_ prior to all experiments to maintain the tissue at close to physiological conditions during electrical measurements.

### Neuroretinal tissue preparation

Eyes from Danish Land Race pigs (*Sus domesticus*) were collected at a slaughterhouse (Danish Crown, Horsens, Denmark) immediately after the animals had been stunned with carbon dioxide and euthanized by exsanguination. The eyes were transported to AUH laboratory in PSS at 4 °C, and the time from the collection of the eyes to the commencement of the dissection procedure never exceeded 1 h. Dissection was performed in 4 °C PSS0.0 as follows: Each eye was bisected at the equator with a double-edged razor blade, and the anterior segment was removed. The posterior segment containing the optical disk was placed under a stereo microscope and the vitreous body was removed. 2–3 mm from the optical disk a segment of approximately 3 mm × 10 mm neuroretina tissue was cut out using a self-locking chisel blade handle (VWR International, Herlev, Denmark) equipped with a 30 microblade (BD Beaver, D.J. Instruments, Billerica, USA). Care was taken to avoid large blood vessels in the selected segment. This study contains data obtained from 10 different eyes from 10 different animals. All tissue samples were thus obtained from different eyes.

### Electrophysiology setup and stimulation

**Figure 1:**
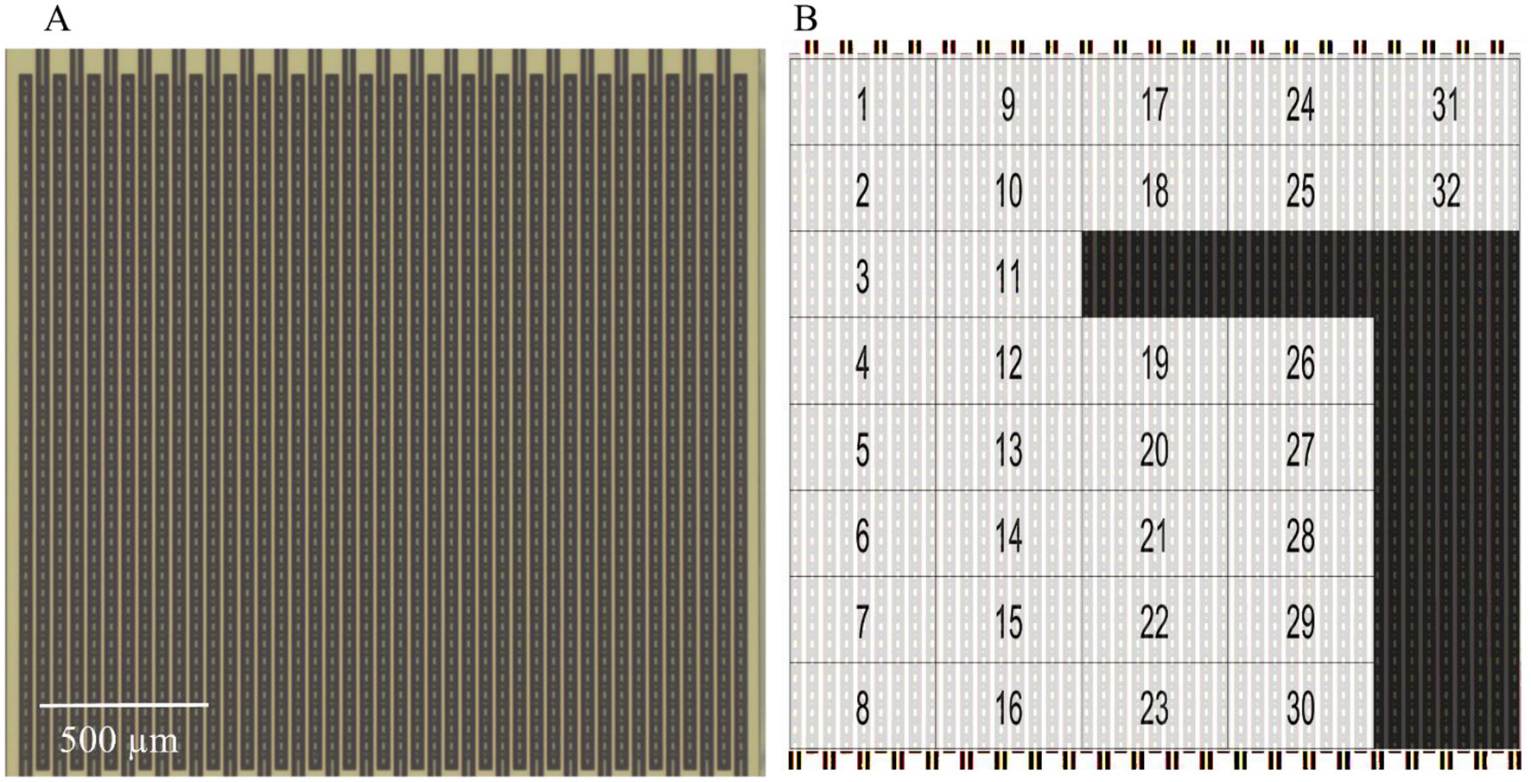
**(A)** Interlaced fingers layout of carbon pillars. **(B)** Layout of MEA channels on top of carbon pillar electrode. Dark shaded area indicates a passive partof the MEA without active channels due to the position of the wiring. The area covered by each channel in the illustration approximates the area surrounding each recording electrode. Each electrode was 80 μm wide at its base, ending in 3–5 μm radius of curvature, with an inter-electrode spacing of 400 μm.

The fabrication process and the characterization of the electrodes have been described previously (Kusumanchi et al., 2025). The carbon electrodes consist of rows of carbon pillars in an interdigitated-finger-design, see Figure 1. Each alternating row of pillars connects to the opposite gold pads on a silicon chip.

For electrophysiological experiments tissue was placed in a 3D printed holder. The tissue samples were placed on the electrode chip with the photoreceptor layer facing down toward the carbon pillar electrodes. A silicone O-ring was placed on top of the chip, with the purpose of sealing the well inside the holder. PSS1.6 was then added to the central reservoir.

A 32 channel Blackrock microelectrode array (MEA) was positioned on the retinal ganglion cell (RGC) side of the tissue through the well after the lid was fastened to the holder bottom (Kusumanchi et al., 2025). A Keysight True Waveform Generator (33500B) was connected to the metal pins in the holder lid, providing contact to the Au pads of the chip and supplying electrical stimulation to the tissue. Data was recorded using the 32 channel Blackrock MEA connected to a Cereplex data acquisition unit (DAQ) via a Cereplex M headstage. The layout of channels in the MEA can be seen in Figure 1. The black void represents the area on the MEA without active channels. Data was sampled at 30 kHz and high pass filtered at 250 Hz before being stored for further analysis. The tissue samples were stimulated by 1 ms pulses of 1 V amplitude, over a −500 mV offset, to provide a net amplitude of +500 mV. The negative offset was applied to avoid positive charge build up. Each stimulation run lasted for 10 s with stimulation at 10 Hz resulting in a total of 100 stimulations.

All stimulation experiments consisted of an initial recording without stimulation to establish the level of spontaneous activity. Subsequently, a recording was performed where the tissue was stimulated as described.

### Data analysis

Data analysis was performed using MATLAB. The high pass filtered data traces were parsed into vectors by identifying the position of stimulation artifacts (SA) throughout the traces. Each stimulation run had a duration of 10 s with a stimulation frequency of 10 Hz. The data was sampled at 30 kHz yielding a total of 100 vectors with 3,000 data points, each starting with an SA. To avoid complications in data analysis caused by the SA at the beginning of each vector, and possibly at the end due to initial effects of the next SA, the vectors were truncated to only contain the datapoints between 450 and 2,650 post the initial SA resulting in a total of 2,200 datapoints pr vector (~73.3 ms). Previous data analysis using this method has described using truncated vectors containing the datapoints between 300 and 2,700 (Kusumanchi et al., 2025). As more replicates have been added in this study, the need for further truncation of the vectors became apparent to avoid complications from the SA.

Evoked compound action potentials (spikes) were counted by identifying values in the truncated vectors which were more than a factor of 4.5 times above the root mean square (RMS) value of the entire vector. Furthermore, to avoid overcounting due to several datapoints of a single spike surpassing this value, a separation of a minimum of 30 datapoints (1 ms) was used before a new value could be counted as a spike. The criterion of 30 data points was based on numerous observations of the duration of evoked spikes. Evoked spikes tended to have returned to baseline after < 30 points.

## Results

**Figure 2:**
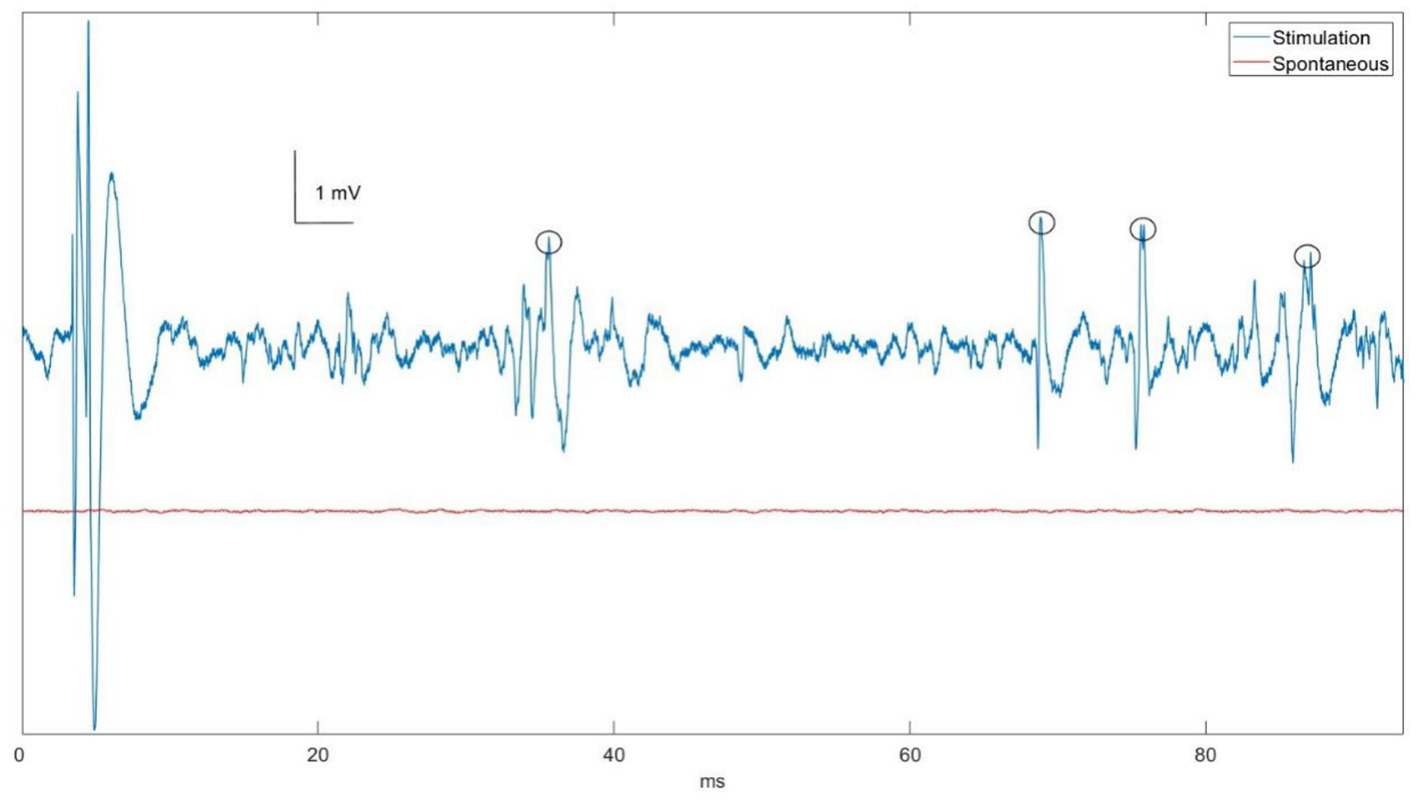
Raw traces from a single MEA channel during stimulation (blue) and spontaneous activity (red). Black circles show spikes with peak amplitudes above 4.5 RMS threshold. The large initial spike represents a stimulus artifact.

Figure 2 shows examples of raw data traces recorded from a single MEA channel during stimulation, stimulation with TTX and spontaneous activity. As can be seen in the trace examples, spikes are more frequent and of an order of magnitude higher amplitude when the tissue is stimulated, compared to a spontaneous recording. Black circles denote spikes with peak values above the 4.5 times RMS threshold which have been counted.

**Figure 3:**
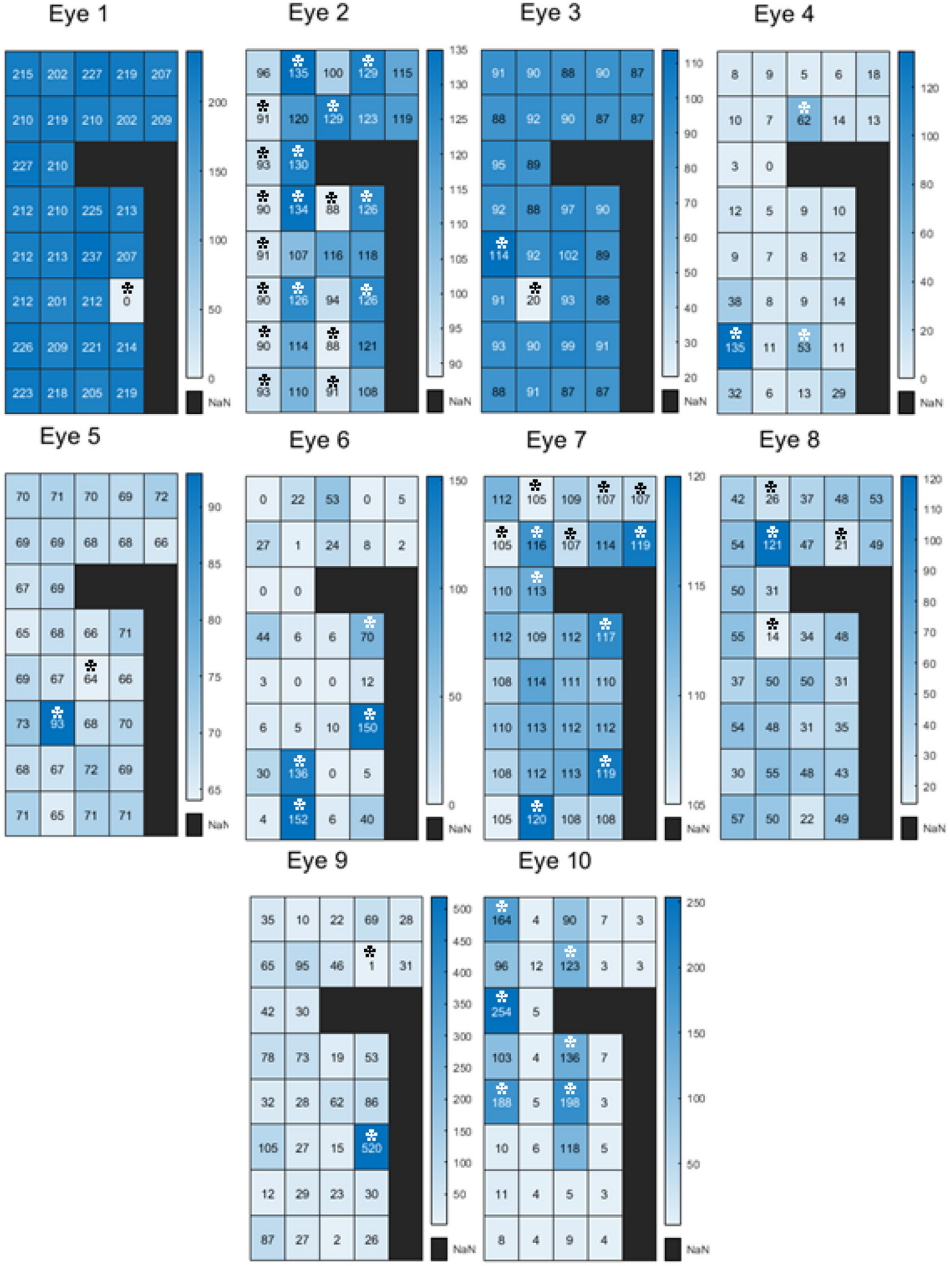
Heatmaps displaying variation of total spikes detected for each MEA channel during stimulation, * denotes channels with total spike counts more than 1 standard deviation from the mean of all channels, white indicates above and black below the mean.

Figure 3 displays a heatmap of the variation of total spikes counted during stimulation for each channel of the 32 channel MEA for all eyes (see Figure 1 for MEA channel layout). The black void in each subfigure in Figure 3 represents the area without recording channels. Asterisks denote channels which have a total spike count that differ by more than one standard deviation from the mean of all channel spike counts for that eye. As can be seen, seven of the 10 eyes displayed five or fewer channels which deviated more than one standard deviation from the mean. We note that both Eyes 4 and 6 have generally low counts, potentially indicating limited stimulation success. Eye 10 also displayed low counts when stimulated, however this was still significantly higher than the spontaneous activity, potentially indicating a generally low excitability of the tissue of Eye 10.

The number of times a given channel displayed spike counts which deviated more than one standard deviation from the mean spike count (Figure 3) were summed in bins corresponding to each channel and tested using one-way-ANOVA to determine if any of the channels displayed a significantly different number of deviations. The test showed no channel was significantly different (*p* = 0.61). Although no channel was significantly different regarding the number of deviations, as can be seen in Figure 3, the inter-eye-variation is substantial. This is the case regarding both spike counts and placement. This variation is likely caused by significant differences in the responsiveness of the tissue and the differences in the setup between samples, such as degree of contact between tissue and electrodes, tissue placement etc. These inherent sources of variation pose specific challenges for these types of measurements. Supplementary Figure S1 summarizes the number of channels with spike counts removed by one standard deviation or more from the mean of each eye.

**Figure 4:**
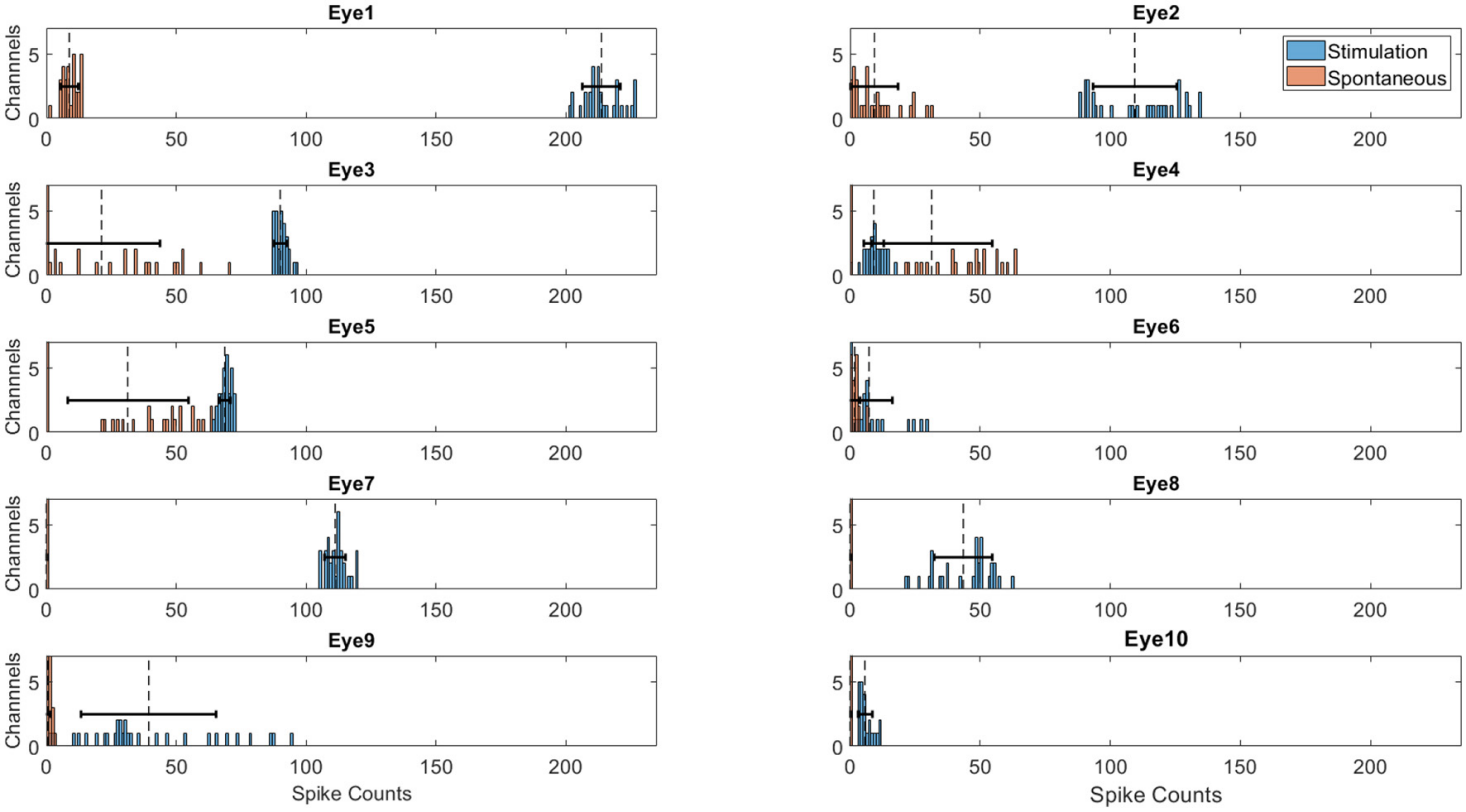
Spike count histograms for stimulation and spontaneous activity for channels for each eye, outliers removed. Vertical lines indicate means spike counts for all channels during stimulation or spontaneous activity, horizontal lines indicate standard deviations.

Figure 4 shows histograms of spike counts for all 32 channels for all eyes (*n* = 10) during stimulation and for spontaneous activity. Dashed vertical lines indicate mean spike counts and horizontal lines indicate standard deviations. All data shown in Figure 4 have outliers removed, an outlier being defined as any value of more than three absolute standard deviations from the median.

Except for Eye 4, there was a significant increase in spike counts (*p* < 0.05) during stimulation (Mann–Whitney *U*-test) for all eyes. As with Figures 3, 4 also shows a large degree of inter-eye-variance both for stimulated and spontaneous spike counts. Some eyes, such 2, 6, 8, and 9 also display relatively large intra-eye-variation in stimulated spike counts between the individual channels, while other eyes, such as 1, 3, 7, and 10 show relatively low intra-eye-variation. Eye 10 displayed a comparatively low number of spikes counted, especially when outliers were removed from both the spontaneous and stimulated count data. The difference between spontaneous and stimulated spike counts for Eye 10 was, as stated above, significant, but only due to the very low spontaneous counts.

Supplementary Figures S2, S3 display data regarding the temporal variation of the recorded spike, both spontaneous and stimulated. While some eyes displayed significant effect of time on spike counts (*p* < 0.01), no discernable pattern was evident in the distribution of spikes across the recording window. Also, the mean spike counts for all eyes across the stimulation window showed no significant effect of time.

## Discussion

The purpose of this study was to analyze the spatial variation of the activity evoked by direct voltage stimulation of retinal tissue using non-metallic 3D pyrolytic carbon electrodes. It is crucial to examine spatial variation of induced responses within a single tissue sample and between several samples, as a pre-evaluation for utilizing such an electrode in a photovoltaic retinal implant. As mentioned, for a successful proof-of-concept, the electrode should (i) be capable of achieving significantly higher tissue activity during stimulation compared to spontaneous activity when supplied with power within the range of a photovoltaic prosthetic device, i.e., ~0.5 V for a single-junction silicon device and (ii) the spatial variation of this evoked activity should display no persistent biases or patterns of activation.

The heat maps shown in Figure 3 give indications of the uniformity of stimulation responses across the different MEA channels. Several eyes, such as eyes 1, 3, and 5, show relatively low variation in the number of counted spikes pr channel, while others, such as eyes 2, 7, and 9, display relatively high variation. Despite this degree of variation, the heat maps shown in Figure 3 also indicate that there does not appear to be any discernable spatial pattern to the variation or effect of the electrode stimulation on the number of spike counts, creating no increased or decreased counts along the periphery or center of the electrode, when looking at tissue activation across different eyes. The lack of spatial bias also confirms that the setup used in this study was adequate to perform the measurements in question. There is variation caused by the different tissue samples used and the degree of contact between tissue and MEA may also cause variation, but the lack of repeated spatial pattern in the evoked activity confirms the setup is unbiased. This is an important point as such a setup is necessary for further studies where heterogenous or pixelated stimulation will be applied to closer mimic natural light stimulation of the retina.

All eyes, except Eye 4, displayed significantly higher spike counts when stimulated, than spontaneous activity, see Figure 4. However, as is also evident in Figure 4, Eyes 6 and 10 have mean stimulated activity levels relatively close to spontaneous, compared with other eyes, such as 1, 3, and 7. Note that all activity levels presented in Figure 4 have outliers removed. This was done to minimize the effect of single outliers, such as channel 28 in Eye 1 showing zero spikes in an otherwise very uniform stimulation, and the same channel for Eye 9, showing 520 spikes (the highest single count) during stimulation with relatively few spikes on all other channels. Removing the outliers avoids undue influence upon the spike counts displayed to give a more accurate comparison of the mean levels of activity during stimulation and spontaneous activity. While it is useful to remove outliers for comparison of the mean activity levels, these channels must still be included when considering whether the evoked activity contained spatial biases. These channels represent large deviations from the mean activity which constitutes uneven stimulation, resulting in possible blind or white spots in the resulting visual information. As the ANOVA testing of the data in Figure 3 determined, no channel had a significant number of deviations from the mean spike count for each individual stimulation, suggesting no spatial pattern or biases in the stimulation. As such, the outliers removed are not considered representative of such spatial biases or patterns.

Taking the data in both Figures 3, 4 into account, it was determined that stimulation activity was significantly above spontaneous activity in nine out of 10 eyes. Furthermore, what could be termed uniform stimulation, defined as spike counts with relatively low variation, of retinal tissue was achieved in Eyes 1, 3, 5, and 7. While other eyes also display clearly increased activity when stimulated, such as 2, 8, and 9, these show high local variation of stimulation responses. Nevertheless, with significantly higher stimulated activity compared with spontaneous, the absence of a spatial pattern in evoked activity, and a demonstrated ability of the electrode to produce uniform tissue activation, at least in some cases, the proof-of-concept of both the electrode and the setup is established. While the ability to achieve uniform activation, i.e., low spatial variation, of tissue when supplied with a direct current pulse is important for the establishment of the proof-of-concept, it could not be expected that the electrode would achieve such activation in all cases.

This study also considered the temporal aspect of the activity evoked by the developed electrode. Supplementary Figures S2, S3 show that, while some eyes did show differences in the variation of spikes across the time of the recording window, no pattern to the timing of responses were found, and the mean spike counts for all eyes did not show any effect of time after stimulation. It is possible that the artificial nature of the setup, with relatively small, excised tissue samples in contact with stimulating and recording electrodes, caused the circumstances which lead to the seemingly randomness of the temporal distribution of spikes post stimulation. It is also possible that the temporal resolution used in the data analysis was insufficient to reveal potential patterns in the temporal variance of the evoked spikes. As is also evident from the data shown in Supplementary Figure S2, the tissue showed no tendency to desensitize or fatigue over time. This is contrary to other findings, which have found retinal neurons tend to desensitize at stimulation frequencies of above 5 Hz (Freeman et al., 2011). This study stimulated at 10 Hz, the lack of desensitization, together with the lack of time locked responses may suggest that different populations of neurons are being activated at different stimulations. For further development of the implant, the temporal aspect of the evoked potential will need to be considered.

An important aspect when considering the future impact of 3D pillar electrodes for vison restoration is the ability to stimulate as locally as possible, preferably only stimulating cells directly in contact with or above the top of each pillar electrode. This will allow for higher resolution of the vision restored. To this end, the analysis developed in this study is important to assess the ability of the carbon electrodes to stimulate locally in future studies. Such technical development is already taking place, with the ability to achieve sidewall passivation of the carbon pillars, isolating and directing the stimulating current to the tip of the pillars only, thereby reducing potential crosstalk in the stimulation of cells by the different pillars (Rezaei et al., 2022).

While many studies exist which attempt to validate newly developed electrodes for retinal stimulation (Khalili Moghadam et al., 2013; Hadjinicolaou et al., 2012; Nayagam et al., 2014; Lohmann et al., 2019), comparatively few of them present any analysis of the spatial variation of the activity they evoke. Some studies do consider spatial interaction to some degree but often limit this to the few RGCs around single electrodes (Sekirnjak et al., 2006; Horsager et al., 2010), rather than whole patches of tissue, which implant electrodes should ultimately stimulate when implanted.

Having established the ability of the electrode to stimulate retinal tissue, the analysis performed in this study represents the next step when trying to achieve artificial restoration of vision, the ability to analyze the spatial variation of the activity evoked. This is an important point as the development of contrast vision and the ability to detect shapes is dependent upon the spatial variation of tissue activation in the retina, corresponding to the visual input. In this study, a uniform current stimulation was used to establish that no bias in spatial activation of tissue existed. In future experiments, utilizing light the resulting spatial variation of tissue activation can be compared to the shape of the visual input.

## Conclusion

This study investigated whether a newly developed 3D pyrolytic carbon electrode could produce unbiased and spatially uniform responses, above spontaneous activity, when stimulating porcine retinal explants. The electrode has been shown to be capable of achieving such stimulation, with all the different sources of variation considered. The goal with this electrode is to incorporate it into an implant and use it *in vivo*. Here, many of the sources of tissue variation discussed would be expected to decrease, as the relevant tissue is still being supported by a living animal.

## Data Availability

The raw data supporting the conclusions of this article will be made available by the authors, without undue reservation.

## Supplementary material

Supplementary Figure 1 Number of channels displaying 1SD or more above (blue) and below (red) the mean spike counts for each eye.

Supplementary Figure 2 Variation in spike counts across 73 ms recording window (2200 datapoints). Each point represents the mean number of spikes counted in that ~7 ms time span (200 datapoints) after stimulation throughout the entire recording session. Bars indicate standard error. Stimulated activity shown in blue, spontaneous in red. Significant effect of time was shown in eyes 1-5 for stimulated activity, none for 6-10. Spontaneous activity showed no significant effect of time.

Supplementary Figure 3 Mean spike counts across all eyes for ~7 ms time spans (200 datapoints). Stimulated spike counts shown in blue, spontaneous in red. Bars indicate standard error. No significant effect was shown for time on stimulated or spontaneous spike counts.
