# Supplementary figures and images for: Spatial variation of evoked potentials in porcine retinas characterized by multi electrode array upon stimulation via 3D pyrolytic carbon electrodes
Source: Front Neurosci. 2026 May 26;20:1808212. doi: 10.3389/fnins.2026.1808212 (PMC13246707; doi:10.3389/fnins.2026.1808212)

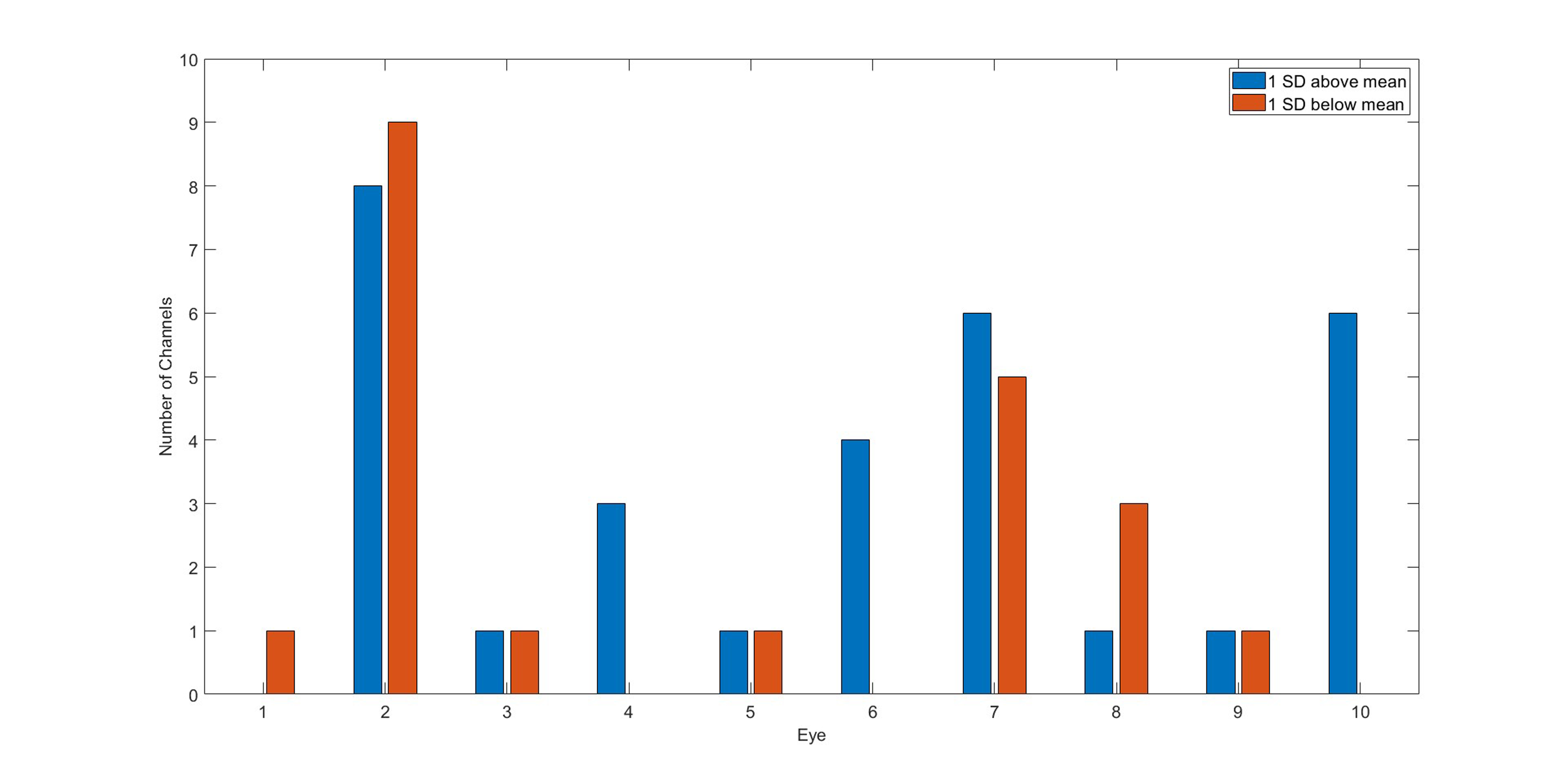

Supplement: Supplementary Figure 1 — Number of channels displaying 1SD or more above (blue) and below (red) the mean spike counts for each eye. [file Image_1.jpeg]

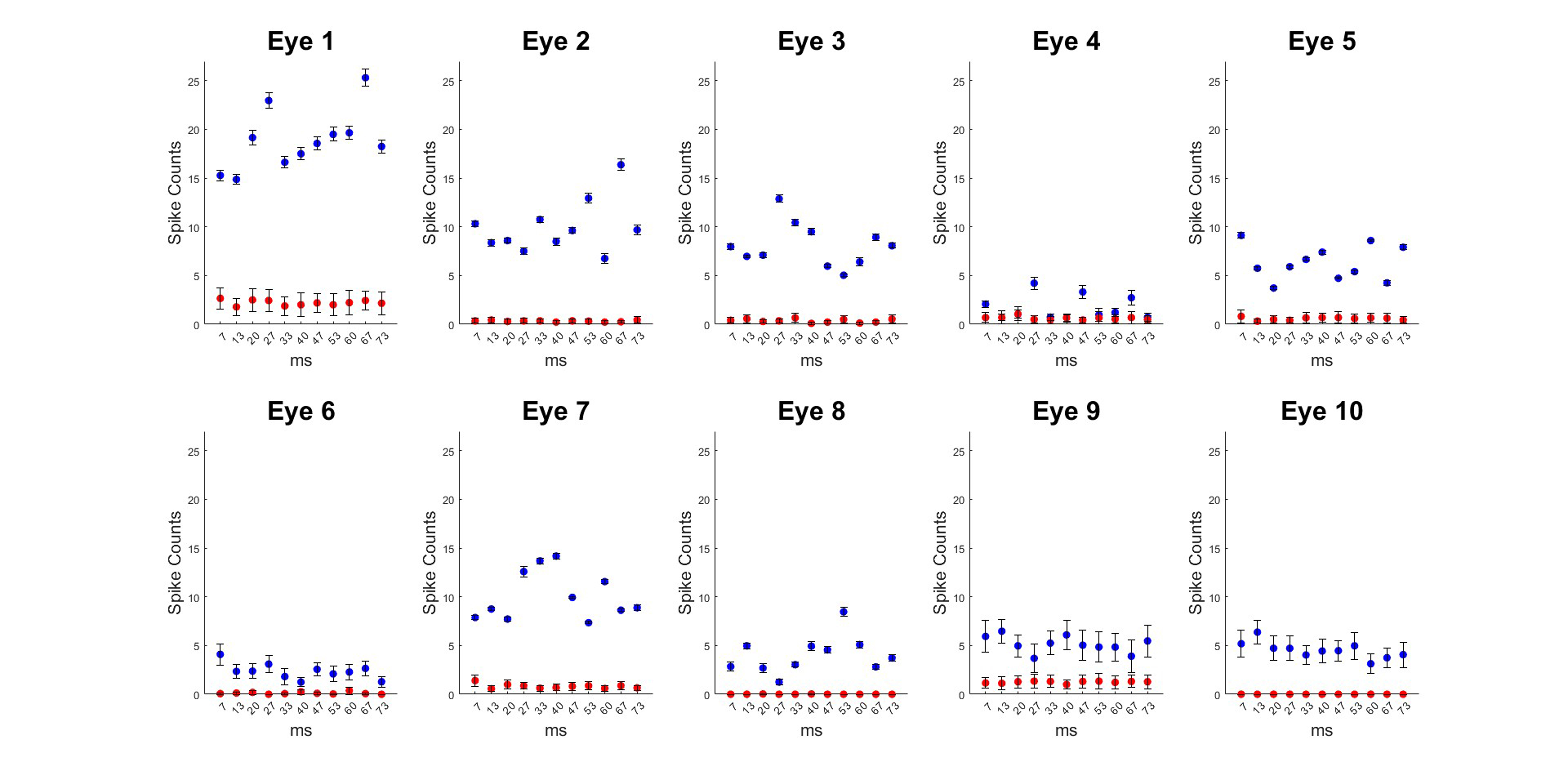

Supplement: Supplementary Figure 2 — Variation in spike counts across 73 ms recording window (2200 datapoints). Each point represents the mean number of spikes counted in that ~7 ms time span (200 datapoints) after stimulation throughout the entire recording session. Bars indicate standard error. Stimulated activity shown in blue, spontaneous in red. Significant effect of time was shown in eyes 1-5 for stimulated activity, none for 6-10. Spontaneous activity showed no significant effect of time. [file Image_2.jpeg]

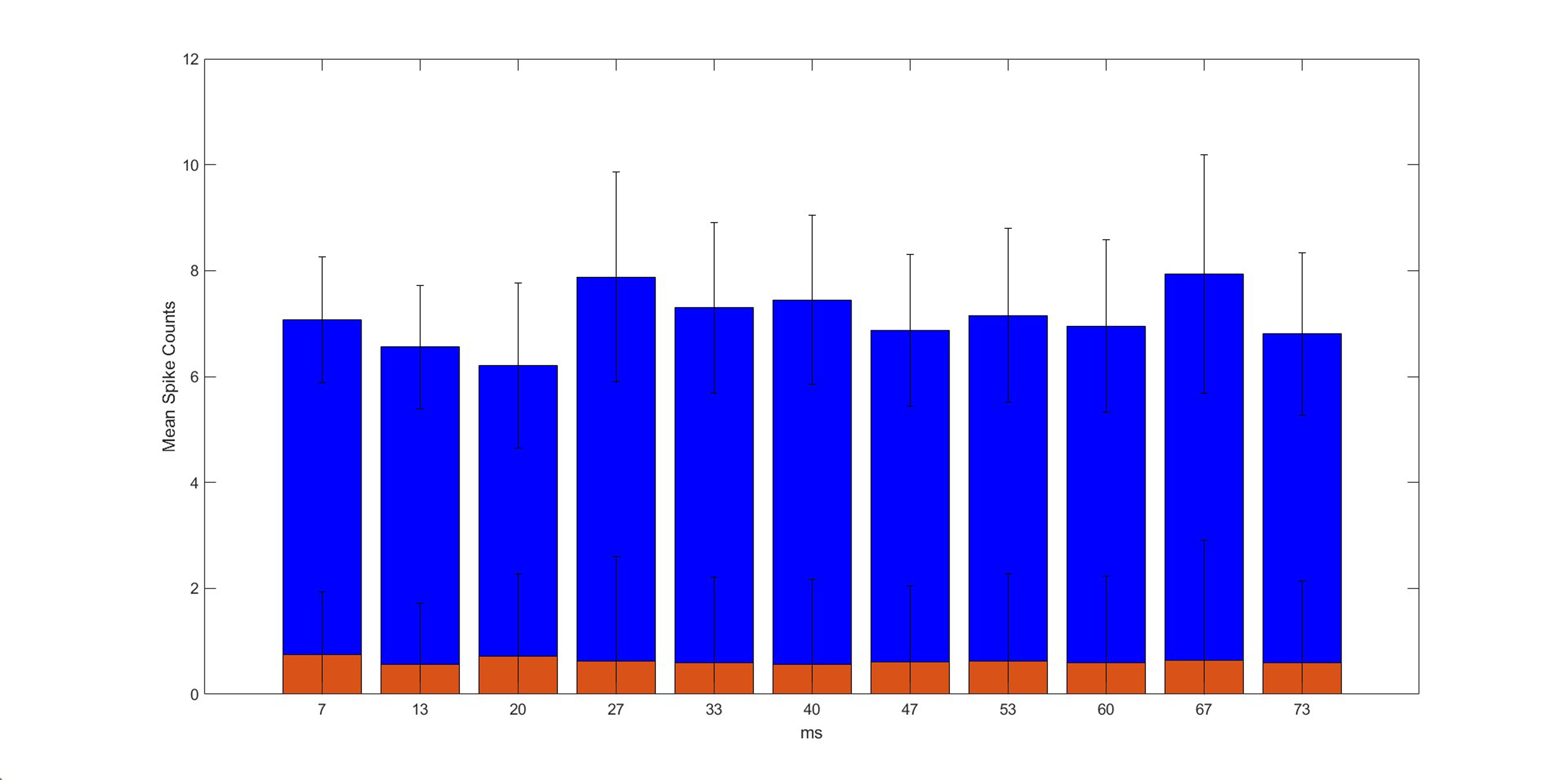

Supplement: Supplementary Figure 3 — Mean spike counts across all eyes for ~7 ms time spans (200 datapoints). Stimulated spike counts shown in blue, spontaneous in red. Bars indicate standard error. No significant effect was shown for time on stimulated or spontaneous spike counts. [file Image_3.jpeg]
